# Evaluation of the effect of intraoperative tropisetron on postoperative rebound pain after brachial plexus block: a randomized controlled trial

**DOI:** 10.1097/PR9.0000000000001163

**Published:** 2024-05-15

**Authors:** Junli Liu, Mingming Liu, Shengnan Shi, Fei Jiang, Ye Zhang, Jing Guo, Xingrui Gong

**Affiliations:** Department of Anesthesiology, Xiangyang Central Hospital, Institution of Neuroscience and Brain Disease, Affiliated Hospital of Hubei University of Arts and Science, Xiangyang, China

**Keywords:** Tropisetron, Analgesia, Rebound pain, Anxiety, Peripheral nerve block

## Abstract

**Introduction:**

Postoperative rebound pain after peripheral nerve block increases patient suffering and delays recovery after surgery.

**Objectives:**

We tested whether the 5HT-3 receptor antagonist and α7nAChR agonist tropisetron could prevent postoperative rebound pain.

**Methods:**

A total of 115 patients were randomized to receive 5-mg/5-mL tropisetron or the same volume of normal saline. Pain intensity was measured with the numerical rating scale of pain (NRS). Rebound pain was defined as a change from mild pain (NRS ≤ 3) measured in the postanesthesia care unit to severe pain (NRS ≥ 7) within 24 hours after peripheral nerve blockade. Logistic regression was used to identify relevant factors associated with postoperative rebound pain.

**Results:**

Tropisetron did not affect the NRS score or the incidence of rebound pain after peripheral nerve block. Logistic regression revealed that preoperative pain, bone surgery, and length of incision were risk factors for postoperative rebound pain, and patient-controlled analgesia was protective against postoperative rebound pain.

**Conclusion:**

Tropisetron does not affect the incidence of rebound pain after peripheral nerve block. Patients at high risk of postoperative rebound pain should be identified for appropriate management. Registration site: www.chictr.org.cn (ChiCTR2300069994).

## Abstract 2

Tropisetron does not affect the incidence of rebound pain; patient-controlled analgesia with opioids could be considered for rebound pain management after peripheral nerve block.

## 1. Background

Orthopedic surgeries are often conducted under peripheral nerve blocks, which are highly effective methods for both anesthesia and analgesia. Peripheral nerve blocks can reduce systemic opioid use and potentially improve patient recovery after surgery.^16^ A single injection can provide analgesic effects for approximately 6 to 8 hours.^1^ However, some patients experience an exaggerated pain response when the block resolves, termed “rebound pain.” Recently, postoperative rebound pain after peripheral nerve block has been widely recognized.^24^ Rebound pain is defined as a transition from well-controlled pain during discharge from the operating room (numerical rating scale of pain [NRS] < 4 on an 11-point scale) to severe pain (NRS ≥7) within 24 hours after the block.^31^ This rebound pain primarily occurs between 12 and 24 hours after the peripheral nerve block is administered.^17,18^ This type of pain is a complex physiological and psychological response that potentially involves multiple factors, such as injection pressure, perineurium edema, myelin degeneration, and local inflammation.^39,43^ In addition, during the surgical procedure, peripheral nerve fibers and bones may be damaged or stimulated because of surgical irritation and tissue injury, resulting in enhanced postoperative pain.^28^ Postoperative rebound pain can decrease a patient's sleep quality, affect their appetite and mood, and diminish their overall recovery. Numerous studies have been conducted to investigate preventive strategies^6,44,46^; however, only a handful of interventions, including steroids and multimodal analgesia, have proven effective.^41^ Despite these measures, the incidence of rebound pain remains considerably high (approximately 20%).^6,45^ Thus, identifying new preventive strategies for this type of pain has become a new challenge for clinicians.^44^

Tropisetron is a 5HT-3 receptor antagonist and partial α7n-type acetylcholine (a7nAch) receptor agonist and has been frequently used for treating postoperative nausea and vomiting (PONV).^36^ Blocking 5HT-3 receptors^35^ and activating a7nAch receptors^26^ produce analgesic effects by decreasing inflammatory cytokine release. Activation of a7nAch receptors has been demonstrated to ameliorate ischemic–reperfusion injury and provide neuroprotection.^32^ In addition, we previously reported that tropisetron reduces inflammation and myocardial ischemic–reperfusion injury after heart valve replacement surgery.^11^ Moreover, antagonism of 5HT-3 receptors has been shown to have anxiolytic effects on animal models through modulation of the serotonergic system.^7,21^ Thus, simultaneously targeting these 2 receptors has the potential to suppress postoperative rebound pain and anxiety. Although many hospitals perform orthopedic surgery as an outpatient procedure, almost all orthopedic surgeries at our hospital involve inpatient surgery, enabling us to observe postoperative rebound pain and anxiety. Thus, we designed this randomized double-blind trial to explore the efficacy of tropisetron on postoperative rebound pain and anxiety after brachial plexus block.

## 2. Methods

The study was approved by the Ethics Committee of Xiangyang Central Hospital affiliated with Hubei University of Arts and Science, and it adhered to the tenets of the Declaration of Helsinki. The clinical study was registered at www.chictr.org.cn (ChiCTR2300069994). Written informed consent was acquired from all patients. The inclusion criteria were as follows: patients aged 18 to 70 years, American Society of Anesthesiologists (ASA) grade I–III anesthesia, and planned to receive upper extremity surgery under brachial plexus block anesthesia from April 2023 to July 2023 in our tertiary hospital. The exclusion criteria were as follows: had cognitive impairment, used other sedative and analgesic drugs in the past week, participated in other drug clinical trials within the past 3 months, or used a 5HT3 receptor antagonist before admission.

Before surgery, a medically trained biostatistician generated randomized numbers with a computer, allocated patients into the control group (5 mL of normal saline) or tropisetron group (5 mg/5 mL of tropisetron), and prepared the drug according to the number sequence. After the patient's admission to an operating room, a noninvasive blood pressure monitor, standard 5-lead electrocardiograph, and pulse oximeter were used for vital sign monitoring. Peripheral nerve block was performed by an experienced attending physician under ultrasound guidance, followed by analgesic effect evaluation 15 minutes after the injection. An anesthesia assistant who was blinded to the group allocation took the drug from the biostatistician and injected it after the start of the surgery. When patients provided informed consent, a patient-controlled analgesia (PCA) pump (100 mL filled with 200 μg of sufentanil) was used for postoperative analgesia. The local anesthetics used were 20 mL of 0.375% ropivacaine and 1% lidocaine for intermuscular sulcus or axillary blocking; 30 mL of 0.375% ropivacaine and 1% lidocaine in total for the intermuscular sulcus (15 mL) and axillary pathway (15 mL); and 30 mL of 0.375% ropivacaine and 1% lidocaine in total for the intermuscular sulcus (20 mL) and cervical plexus blocking (10 mL). If a patient felt pain and could not endure the incision or had an NRS > 3 during the surgery, the patient was considered to have an inadequate block. The recovery of sensory and motor functions was reported by the patients 24 hours after the brachial plexus block. Postoperatively, patients were educated to either press the PCA button or call for a physician if their NRS was equal to or higher than 4. The attending physician would then prescribe parenteral nonsteroid anti-inflammatory drugs (NSAIDs). In cases where the NRS remained ≥ 4, even 30 minutes after receiving the NSAID, an intravenous dose of dezocine was administered.

The demographic information of all the patients was collected, including age, sex, weight, height, smoking status, alcohol consumption, previous disease (cerebrovascular disease, cardiovascular disease, hepatic disease, pulmonary disease, diabetes mellitus [DM], and hypertension), pain before surgery, ASA classification, heart function (New York Heart Association standard), white blood cell (WBC) count, surgery site, bone surgery, length of incision, anesthesia injection site, intraoperative opioid use, intraoperative NSAIDs, dexmedetomidine, and PCA use. The patients' previous diseases were diagnosed through a combination of self-reported history and computed X-ray tomography, magnetic resonance imaging, blood tests, electrocardiography, and angiography. Our study's primary outcome was the incidence of postoperative rebound pain. The secondary outcomes, including visual analog scale of anxiety (VAA), postoperative NSAID use, opioid requirement, duration of sensory block and motor block (recorded in hours), inadequate blocking, postoperative nausea and vomiting (PONV), vertigo, and patient satisfaction, were measured 24 hours after the peripheral block was administered. The NRS scores and other secondary outcomes were measured by an assistant observer unaware of the group allocations. We used the NRS, an 11-point pain intensity score (0–10), to evaluate patient pain intensity, following our previous study. On this scale, “0” signifies no pain, and “10” represents the most intense pain imaginable.^15^ We also used the VAA, another 11-point score, to assess postoperative patient anxiety,^25,47^ as per our previous study.^50^ A VAA score of “0,” ≤3, ≥4, and <7 or ≥7 indicated no, mild, moderate, or severe anxiety, respectively. An independent investigator blinded to the group allocation recorded the patient demographic information and postoperative evaluations. All surgeons, anesthetists, patients, and postoperative evaluators were blinded to the group allocations.

### 2.1. Statistical analysis

The sample size calculation used PASS11 software. Based on a previous study, the incidence of rebound pain is about 50% after peripheral nerve block, and a 0.2 decrease of incidence is considered significant. The sample size was at least 39 patients in each group, with an alpha of 0.05 and a beta of 0.20.^44^ Considering that approximately 30% of patients are lost to follow-up, we included 115 patients in total. Continuous data are presented as the mean (SD) or median (interquartile) if they were normally or nonnormally distributed; 2 groups were compared using *t* tests or Mann–Whitney *U* tests. The categorical data are presented as numbers and tested using the χ^2^ or Fisher exact tests. The comparisons between groups were performed through intention-to-treat analysis. Univariate and multivariate logistic regression analyses were performed to explore the risk factors for postoperative rebound pain and moderate to severe anxiety (VAA ≥4). A *P* value <0.05 was considered to indicate a significant difference.

## 3. Results

### 3.1. Patients' baseline characteristics

**Figure 1.**
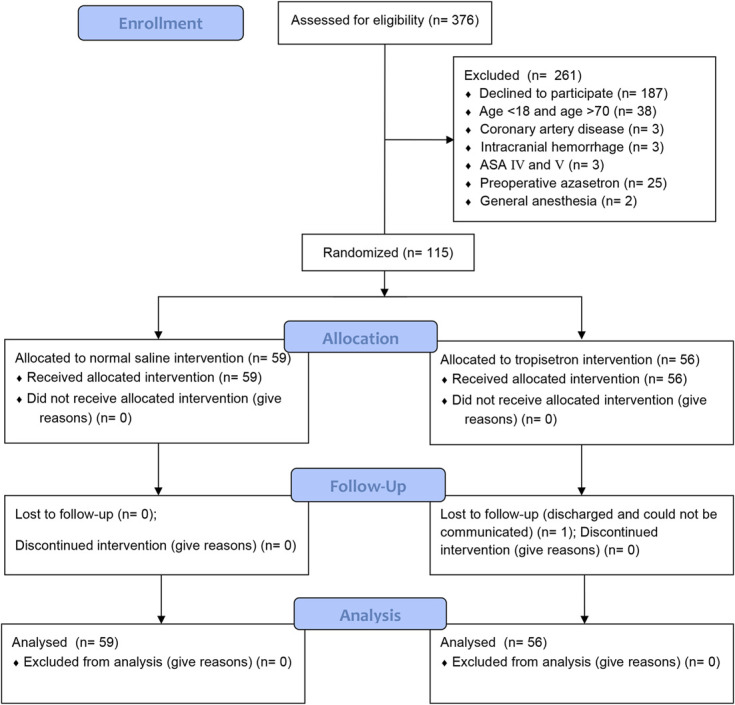
Trial flowchart.

During the study period, we screened a total of 376 patients and enrolled 115 patients. Among those patients, 56 (33 men) and 59 (34 men) patients were randomized to the tropisetron and saline groups, respectively. The trial flow chart is shown in Figure 1. None of the anesthesia procedures were converted to general anesthesia. One patient in the tropisetron group was discharged before the first visit and could not be contacted. The 2 groups showed no significant differences regarding demographic information, history, or NRS score before surgery (Table 1). There was no significant difference between the 2 groups regarding surgery-related information, including surgery location, bone surgery, or incision length (Table 1). In addition, no significant differences were found between the 2 groups regarding anesthesia-related information, including injection site, inadequate blocks, intraoperative NSAID, dexmedetomidine, or PCA use (Table 1). One patient reported an inadequate block; he could not endure the incision and had an NRS score >3 during surgery. The patient subsequently received sufentanil and dexmedetomidine for analgesia and sedation, respectively.

**Table 1:** Patient baseline characteristics.

	Control (n = 59)	Tropisetron (n = 56)	*P*
Women (%)	26 (44.1)	22 (39.3)	0.741
Age (mean [SD])	50.08 (14.24)	48.23 (15.19)	0.501
Weight (mean [SD])	66.69 (11.59)	66.32 (12.47)	0.868
Height (mean [SD])	165.81 (7.86)	165.36 (6.72)	0.739
BMI (mean [SD])	24.16 (3.14)	24.15 (3.61)	0.989
Smokers (%)	10 (16.9)	5 (8.9)	0.318
Alcohol use (%)	20 (33.9)	15 (26.8)	0.531
Pain before surgery (median [IQR])	5.00 (3.00–5.00)	4.00 (2.00–5.00)	0.282
Cerebrovascular disease yes (%)	4 (6.8)	5 (8.9)	0.935
Cardiovascular disease yes (%)	0 (0.0)	1 (1.8)	0.979
Pulmonary disease yes (%)	2 (3.4)	5 (8.9)	0.394
Hypertension yes (%)	10 (16.9)	11 (19.6)	0.895
DM (%)	3 (5.1)	3 (5.4)	1
Hepatic disease no/yes (%)	4 (6.8)	2 (3.6)	0.723
ASA (%)			0.4
II	48 (81.4)	41 (73.2)	
III	11 (18.6)	15 (26.8)	
Heart function (%)			0.897
I	4 (6.8)	3 (5.4)	
II	51 (86.4)	50 (89.3)	
III	4 (6.8)	3 (5.4)	
WBC (mean (SD))	7.21 (2.60)	7.31 (2.97)	0.846
Surgery site (%)			0.796
Hand	14 (23.7)	12 (21.4)	
Front arm	25 (42.4)	24 (42.9)	
Elbow	2 (3.4)	5 (8.9)	
Upper arm	17 (28.8)	14 (25.0)	
Shoulder	1 (1.7)	1 (1.8)	
Bone surgery (%)	47 (79.7)	38 (67.9)	0.219
Length of incision (median [IQR])	7.00 (4.00–10.00)	10.00 (4.75–10.00)	0.324
Anesthesia injecting site (%)			0.718
Intermuscular sulcus	19 (32.2)	16 (28.6)	
Axillary pathway	3 (5.1)	1 (1.8)	
Intermuscular sulcus + axillary pathway	26 (44.1)	26 (46.4)	
Intermuscular sulcus + cervical plexus	11 (18.6)	13 (23.2)	
Inadequate block (n)	0	1	0.979
Intraoperative opioid use (%)	30 (50.8)	31 (55.4)	0.766
Intraoperative NSAID use (%)	10 (16.9)	9 (16.1)	1
Intraoperative dexmedetomidine use (%)	58 (98.3)	55 (98.2)	1
PCA yes (%)	7 (11.9)	3 (5.4)	0.365

BMI, body mass index; DM, diabetes mellitus; NSAIDs, nonsteroidal anti-inflammatory drugs; PCA, patient-controlled analgesia; WBC, white blood cell count.

### 3.2. Postoperative primary and secondary outcomes

The intention-to-treat analysis of the primary outcomes revealed no significant differences between the tropisetron and saline groups regarding the incidence of rebound pain (tropisetron, 62.7%; control, 55.4%, *P* = 0.487) or NRS score (tropisetron, 7.00 [4.00, 8.00]; control, 7.00 [5.00, 8.00], *P* = 0.539) 24 hours after surgery (Table 2). However, the postoperative VAA score (tropisetron, 2.00 [1.00, 3.00]; control, 3.00 [2.00, 5.00], *P* = 0.006) and incidence of moderate to severe anxiety (tropisetron, 21.4%; control, 44.1%; *P* = 0.017) were significantly lower in the tropisetron group than those in the control group. The postoperative durations of sensory (tropisetron, 8.00 [7.00, 10.00]; control, 8.00 [7.00, 9.00], *P* = 0.860) and motor (tropisetron, 8.00 [7.00, 9.25]; control, 8.00 [7.00, 9.00], *P* = 0.997) blockages did not differ between the 2 groups. Similarly, the use of postoperative analgesia with NSAIDs (tropisetron, 96.4%; control, 100%; *P* = 0.453) and opioids (tropisetron, 8.9%; control, 8.5%; *P* = 1; did not include patient-use PCA) did not differ between the 2 groups. Moreover, there were no significant differences in terms of postoperative adverse events, including PONV (*P* = 0.113), postoperative headache (tropisetron, 0%; control, 3.4%; *P* = 0.499), or vertigo (tropisetron, 1.8%; control, 11.9%; *P* = 0.079). Importantly, the patients' analgesia satisfaction scores were similar between the 2 groups (*P* = 0.714).

**Table 2:** Evaluation of postoperative primary and secondary outcomes.

	Control (n = 59)	Tropisetron (n = 56)	*P*
Highest NRS within 24 hours after block (median [IQR])	7.00 (5.00–8.00)	7.00 (4.00–8.00)	0.539
Rebound pain present (%)	37 (62.7)	31 (55.4)	0.487
Postoperative NSAID use (%)	59 (100.0)	54 (96.4)	0.453
Postoperative opioid use (%)	5 (8.5)	5 (8.9)	1
Duration of sensory block (median [IQR])	8.00 (7.00–9.00)	8.00 (7.00–10.00)	0.860
Duration of motor block (median [IQR])	8.00 (7.00–9.00)	8.00 (7.00–9.25)	0.977
VAA (median [IQR])	3.00 (2.00–5.00)	2.00 (1.00–3.00)	0.006
Moderate to severe VAA (%)	26 (44.1)	12 (21.4)	0.017
Postoperative nausea and vomiting (%)			0.113
No	52 (88.1)	41 (73.2)	
Mild nausea	5 (8.5)	9 (16.1)	
Nausea and mild vomiting	2 (3.4)	6 (10.7)	
Analgesia satisfaction (%)			0.714
Medium	1 (1.7)	2 (3.6)	
Satisfied	24 (40.7)	25 (44.6)	
Very satisfied	34 (57.6)	29 (51.8)	
Postoperative headache (%)	2 (3.4)	0 (0.0)	0.499
Postoperative vertigo (%)	7 (11.9)	1 (1.8)	0.079

NSAIDs, nonsteroidal anti-inflammatory drug; NRS, numerical rating scale of pain; VAA, visual analog scale of anxiety.

### 3.3. Identification of factors associated with postoperative rebound pain and moderate to severe anxiety

Of the 115 patients, 114 had complete data, and the treatment and normal saline group patients' data were combined and used for associated factor analysis. Univariate logistic regression analysis revealed that preoperative pain, WBC count, bone surgery, and incision length were risk factors for postoperative rebound pain, and PCA was protective against this pain. Multivariate logistic regression analysis revealed that preoperative pain (4.8 [2.62, 10.93], bone surgery (8.5 [1.66, 56.32]), and incision length (1.6 [1.28, 2.13]) were risk factors, and PCA (0 [0, 0.04]) was protective against postoperative rebound pain (Table 3).

**Table 3:** Univariate and multivariate logistic regression analyses of risk factors for postoperative rebound pain.

	No rebound pain (n = 47)	With rebound pain (n = 67)	*P* Univariate	*P* Multivariate	OR (95% CI)
Tropisetron use (%)	25 (53.2)	30 (44.8)	0.487		
Women (%)	15 (31.9)	33 (49.3)	0.098		
Age (mean [SD])	49.15 (14.00)	49.28 (15.33)	0.962		
Weight (mean [SD])	68.06 (10.90)	65.01 (12.23)	0.174		
Height (mean [SD])	166.34 (6.47)	164.97 (7.84)	0.326		
BMI (mean [SD])	24.52 (3.02)	23.79 (3.47)	0.25		
Smokers (%)	5 (10.6)	10 (14.9)	0.7		
Alcohol use (%)	17 (36.2)	17 (25.4)	0.302		
Pain before surgery (median [IQR])	2.00 (1.00–3.00)	5.00 (4.00–5.00)	0.001	<0.001	4.8 (2.62–10.93)
Cerebrovascular disease (%)	4 (8.5)	5 (7.5)	1		
Cardiovascular disease (%)	0 (0.0)	1 (1.5)	1		
Pulmonary disease (%)	2 (4.3)	4 (6.0)	1		
Hypertension (%)	8 (17.0)	13 (19.4)	0.938		
DM (%)	2 (4.3)	3 (4.5)	1		
Hepatic disease (%)	2 (4.3)	4 (6.0)	1		
ASA (%)			0.343		
II	34 (72.3)	54 (80.6)			
III	13 (27.7)	13 (19.4)			
Heart function (%)			0.094		
I	5 (10.6)	2 (3.0)			
II	41 (87.2)	59 (88.1)			
III	1 (2.1)	6 (9.0)			
WBC (mean [SD])	6.58 (2.99)	7.75 (2.55)	0.027	0.345	0.89 (0.69–1.13)
Surgery site (%)			0.572		
Hand	10 (21.3)	16 (23.9)			
Front arm	19 (40.4)	29 (43.3)			
Elbow	5 (10.6)	2 (3.0)			
Upper arm	12 (25.5)	19 (28.4)			
Shoulder	1 (2.1)	1 (1.5)			
Bone surgery (%)	22 (46.8)	63 (94.0)	<0.001	0.015	8.5 (1.66–56.32)
Length of incision (median [IQR])	4.00 (2.00–10.00)	10.00 (6.00–10.00)	<0.001	<0.001	1.6 (1.28–2.13)
Anesthesia site (%)			0.257		
Intermuscular sulcus	19 (40.4)	16 (23.9)			
Axillary pathway	2 (4.3)	2 (3.0)			
Intermuscular sulcus + axillary pathway	17 (36.2)	34 (50.7)			
Intermuscular sulcus + cervical plexus	9 (19.1)	15 (22.4)			
Intraoperative opioid (not used/used) (%)	19/28 (40.4/59.6)	34/33 (50.7/49.3)	0.37		
Intraoperative NSAID use (%)	11 (23.4)	8 (11.9)	0.173		
Intraoperative dexmedetomidine use (%)	46 (97.9)	66 (98.5)	1		
PCA use (%)	8 (17.0)	2 (3.0)	0.023	<0.001	0 (0–0.04)
Postoperative NSAID use (%)	46 (97.9)	67 (100.0)	0.858		
Postoperative opioid use (%)	3 (6.4)	7 (10.4)	0.675		

BMI, body mass index; DM, diabetes mellitus; NSAIDs, nonsteroidal anti-inflammatory drugs; PCA, patient-controlled analgesia; WBC, white blood cell count.

**Figure 2.**
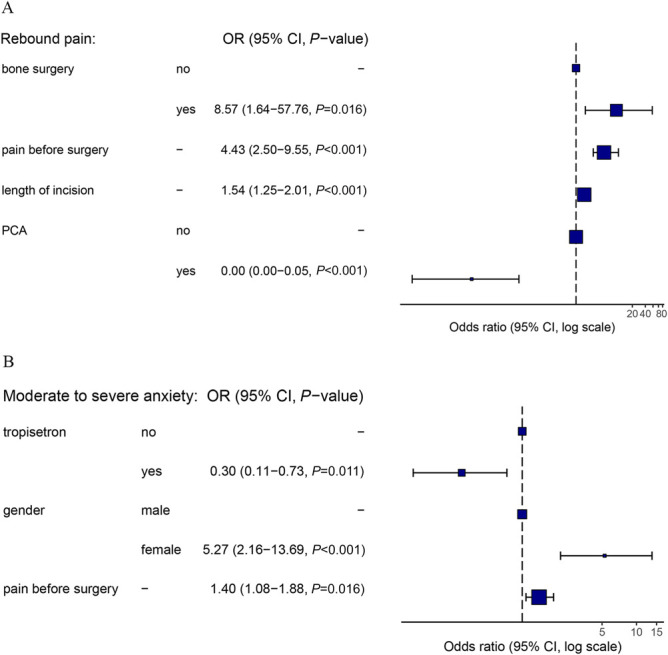
Forest plots of the relevant factors of (A) rebound pain and (B) moderate to severe anxiety after brachial plexus block.

Univariate logistic regression analysis revealed that tropisetron use, female sex, age, height, alcohol consumption, and preoperative pain were associated with postoperative moderate to severe anxiety. Multivariate logistic regression analysis revealed that female sex (4.91 [1.99, 12.93]) and preoperative pain (1.37 [1.05, 1.84]) were risk factors, and tropisetron (0.29 [0.11, 0.75]) was protective against postoperative moderate to severe anxiety (Table 4). The forest plots for the relevant factors of rebound pain and moderate to severe anxiety are presented in Figure 2A and 2B, respectively.

**Table 4:** Univariate and multivariate logistic regression analyses of risk factors for postoperative moderate to severe anxiety.

	No to mild anxiety (n = 77)	Moderate to severe anxiety (n = 37)	*P* Univariate	*P* Multivariate	OR (95% CI)
Tropisetron use (%)	44 (57.1)	11 (29.7)	0.011	0.012	0.29 (0.11–0.75)
Women (%)	23 (29.9)	25 (67.6)	0.001	0.013	4.91 (1.99–12.93)
Age (mean [SD])	47.05 (15.30)	53.76 (12.49)	0.022	0.087	1.02 (0.97–1.07)
Weight (mean [SD])	67.32 (11.65)	64.08 (11.82)	0.169		
Height (mean [SD])	166.49 (7.06)	163.54 (7.49)	0.043	0.321	
BMI (mean [SD])	24.21 (3.33)	23.84 (3.26)	0.576		
Smokers (%)	11 (14.3)	4 (10.8)	0.827		
Alcohol use (%)	28 (36.4)	6 (16.2)	0.047	0.359	
Pain before surgery (median [IQR])	3.00 (2.00–5.00)	5.00 (4.00–5.00)	0.003	0.035	1.37 (1.05–1.84)
Cerebrovascular disease (%)	6 (7.8)	3 (8.1)	1		
Cardiovascular disease (%)	1 (1.3)	0 (0.0)	1		
Pulmonary disease (%)	3 (3.9)	3 (8.1)	0.621		
Hypertension (%)	11 (14.3)	10 (27.0)	0.166		
DM (%)	4 (5.2)	1 (2.7)	0.905		
Hepatic disease (%)	3 (3.9)	3 (8.1)	0.621		
ASA (%)			0.726		
II	60 (77.9)	28 (75.7)			
III	17 (22.1)	9 (24.3)			
Heart function (%)			0.352		
I	5 (6.5)	2 (5.4)			
II	69 (89.6)	31 (83.8)			
III	3 (3.9)	4 (10.8)			
WBC (mean [SD])	7.41 (2.84)	6.96 (2.69)	0.417		
Surgery site (%)			0.933		
Hand	19 (24.7)	7 (18.9)			
Front arm	32 (41.6)	16 (43.2)			
Elbow	5 (6.5)	2 (5.4)			
Upper arm	20 (26.0)	11 (29.7)			
Shoulder	1 (1.3)	1 (2.7)			
Bone surgery (%)	53 (68.8)	32 (86.5)	0.072		
Length of incision (median [IQR])	8.00 (4.00–10.00)	10.00 (5.00–10.00)	0.388		
Anesthesia site (%)			0.712		
Intermuscular sulcus	24 (31.2)	11 (29.7)			
Axillary pathway	2 (2.6)	2 (5.4)			
Intermuscular sulcus + axillary pathway	33 (42.9)	18 (48.6)			
Intermuscular sulcus + cervical plexus	18 (23.4)	6 (16.2)			
Intraoperative opioid use (%)	43 (55.8)	18 (48.6)	0.603		
Intraoperative NSAID use (%)	14 (18.2)	5 (13.5)	0.72		
Intraoperative dexmedetomidine use (%)	76 (98.7)	36 (97.3)	1		
PCA (not used/used) (%)	7 (9.1)	3 (8.1)	1		
Postoperative NSAID use (%)	76 (98.7)	37 (100.0)	1		
Postoperative opioid use (%)	6 (7.8)	4 (10.8)	0.857		

BMI, body mass index; DM, diabetes mellitus; NSAIDs, nonsteroidal anti-inflammatory drugs; PCA, patient-controlled analgesia; WBC, white blood cell count.

## 4. Discussion

Our study showed that intraoperative tropisetron did not affect the incidence of postoperative rebound pain. Multivariate logistic regression analysis revealed that preoperative pain, bone surgery, and incision length were risk factors for postoperative rebound pain, and the use of a PCA pump with opioids was protective against postoperative rebound pain.

Previous studies have demonstrated that inhibiting 5-HT3Rs or activating a7nAch receptors has analgesic and neuroprotective effects.^13,30^ 5HT3 receptors are ligand-gated cation channels, and activation of these receptors results in pain signal transmission and central sensitization.^12,19,20^ In addition to the findings in animal models, the analgesic effects of 5-HT3 receptor antagonists on pain in the clinic have been widely explored. Postoperative pain is the most common pain symptom, and 5-HT3 receptor antagonists have been demonstrated to provide effective analgesia for this type of pain. One randomized trial demonstrated that the addition of ondansetron increases the analgesic effect of paracetamol, reduces the postoperative analgesic requirement, and improves patients' postoperative comfort.^8^ Another randomized double-blinded placebo-controlled trial demonstrated that ondansetron is beneficial for the control of pain and withdrawal symptoms after general anesthesia in opioid-addicted patients.^37^ In addition, a double-blind, placebo-controlled trial demonstrated that the addition of intraperitoneal ondansetron decreased the area under the response curve (AUC) of visual analog scores of pain and rescue analgesic requirements compared with those of the control group in patients who underwent laparoscopic cholecystectomy.^2^ Furthermore, a single-dose intravenous injection of the 5HT3 receptor antagonist tropisetron after anesthesia induction significantly reduces postoperative pain scores in patients undergoing gynecological laparoscopies under sevoflurane-based general anesthesia.^38^ However, a recent study demonstrated that tropisetron is ineffective for chronic pain, the reason for which is unknown.^42^ Our study also showed that tropisetron did not decrease the pain score or incidence of postoperative rebound pain after peripheral nerve block. The reasons for this may be as follows. First, tropisetron is metabolized by the liver cytochrome P450 2D6 enzyme system. The mean elimination half-life after intravenous administration is 7 hours, despite the antiemetic effect lasting more than 24 hours.^33^ This suggests that the absence of analgesia might be because the emergence of rebound pain occurs mostly within 12 to 24 hours after the block is administered, while the peak analgesic effect of tropisetron occurs within 12 hours. Secondarily, the analgesic effect of 5-mg tropisetron may be too mild to show a significant difference postoperatively. Finally, these different results may be attributable to differences in the type of 5HT3 antagonist, type of surgery, dose, or outcome. Thus, continuously administering a larger dose of tropisetron for at least 24 hours may demonstrate a better analgesic effect and provide more robust analgesic effects.

Several studies have been conducted on the occurrence and prevention of rebound pain after peripheral nerve block, providing valuable insights into this phenomenon. For instance, Garrett S. Barry et al.^6^ conducted a retrospective study and reported that age, female sex, bone surgery, and dexamethasone use were associated with postoperative rebound pain after peripheral nerve blocks. Several randomized controlled trials further confirmed the effectiveness of dexamethasone.^45,46^ The authors found that the addition of dexamethasone significantly reduced rebound pain compared with that in the control group. The reasons for this may be that dexamethasone reduces surgical trauma-induced immune cell mobilization, cytokine production, and the proinflammatory response cascade.^10^ However, the incidence of rebound pain remains very high (approximately 20%), and clinicians do not use dexamethasone to prevent rebound pain considering the side effects of increased risk of infection or rising blood sugar level in diabetic patients. Thus, future studies should be conducted to explore additional preventive strategies to suppress postoperative rebound pain.

Our study identified 4 factors associated with rebound pain after peripheral nerve block: preoperative pain, bone surgery, incision length, and patient-controlled analgesia. Preoperative pain has consistently been identified as a risk factor for postoperative pain in various surgical settings.^49^ Higher preoperative pain levels may indicate more extensive tissue damage, leading to increased inflammation and sensitization of peripheral nerves. This heightened sensitivity can contribute to the development of rebound pain after peripheral nerve block. Bone surgery, such as orthopedic procedures, often involves more extensive tissue trauma and inflammation than soft-tissue surgeries. The presence of hardware, such as screws or plates, also causes persistent local tissue inflammation after surgery.^5,9^ The release of inflammatory mediators and the activation of nociceptive pathways can contribute to the development of rebound pain. Surprisingly, surgery location is not as important as surgery type in the context of rebound pain, and the reason for this difference is unknown. The incision length is another important factor to consider. Longer incisions may result in increased tissue trauma and disruption of a larger area, leading to a greater likelihood of rebound pain. The extent of tissue damage and subsequent inflammation can influence the severity and duration of rebound pain. Interestingly, our results suggested that the use of PCA was associated with a reduced risk of rebound pain. PCA allows patients to self-administer analgesics, providing timely pain relief and potentially minimizing the occurrence of rebound pain. The ability to titrate analgesics may help maintain a more stable analgesic effect and reduce the risk of rebound pain after peripheral nerve block.

In our study, patients in the tropisetron group had a lower postoperative anxiety score and a lower incidence of moderate to severe anxiety who underwent peripheral nerve block. The median VAA scores in the study are not very high, which suggests that most patients experience low levels of anxiety after surgery. Yet, it is crucial to note that the incidence of moderate to severe anxiety, an indicator for medical intervention, differs between the 2 groups. These findings suggest a potential role for tropisetron in managing perioperative anxiety. The mechanisms underlying the anxiolytic effects of tropisetron may involve interactions with various neurotransmitter systems.^3^ Tropisetron is an analog of 5HT, a neurotransmitter known to regulate mood and anxiety. Increased serotonin levels in the brain can promote feelings of well-being and relaxation. Tropisetron supplementation may enhance serotonin synthesis, leading to reduced anxiety in the perioperative period.^40^ In addition, tropisetron has been shown to modulate the gamma-aminobutyric acid (GABA) system, which plays a crucial role in anxiety regulation.^27^ GABA is an inhibitory neurotransmitter that promotes relaxation and reduces anxiety, and tropisetron can increase GABA levels in the brain, exerting anxiolytic effects. Furthermore, 1 study demonstrated that tropisetron attenuates the anxiogenic effects of social isolation stress by mitigating the negative effects of nitric oxide on mitochondrial function.^22^ Finally, tropisetron did not decrease the incidence of PONV between the study and control groups in our study. This finding may be due to the low incidence of PONV. Another possible explanation could be the greater number of patients who smoked and consumed alcohol in the control group. Although the difference was not statistically significant, both smoking^4^ and alcohol consumption^29,48^ habits are associated with decreased risk of PONV and may have resulted in the lack of difference between the 2 groups.

Our regression analysis identified 3 factors associated with postoperative moderate to severe anxiety: female sex, preoperative pain, and tropisetron administration. Female sex has consistently been reported as a risk factor for increased anxiety in various clinical settings.^14,23^ Hormonal and psychosocial factors may contribute to the higher incidence of anxiety in women.^34^ Preoperative pain was also identified as a risk factor for postoperative anxiety. Patients with higher preoperative pain levels may experience heightened anxiety because of the anticipation of postoperative pain and uncertainty about pain management. Adequately addressing preoperative pain through multimodal analgesia strategies may help mitigate anxiety in these patients. Notably, anxiety is a complex and multifactorial phenomenon, and additional research is needed to further elucidate the potential interventions for anxiety management in the perioperative setting.

This study has several limitations. First, this was a single-center study, and we tested the effects of tropisetron in patients who underwent arm surgery; however, whether it is effective for preventing rebound pain during other surgeries remains to be studied. In addition, our study used 5 mg of tropisetron for treating postoperative rebound pain, but whether a higher dose of 5 mg of tropisetron combined with a continuous administration regimen is effective remains to be determined. Second, we did not measure the baseline anxiety score, which could complicate our understanding of the anxiolytic effect of tropisetron. This lack of baseline data may have prevented us from accurately assessing the degree of anxiety reduction attributable to tropisetron, as we could not compare postoperative scores to preoperative levels. Finally, we did not monitor the pain score at regular intervals (eg, hourly, 2-hourly, or even 4-hourly) during the first 24 hours after surgery. Patient recall of their highest pain score and when the block (motor and sensory) wore off may have led to inaccurate data. Generally, the analgesic effect of opioids relies on 5HT release in the spinal cord, and the addition of a 5HT3 receptor antagonist reduces opioid-induced hyperalgesia and tolerance.^35^ Thus, in the future, conducting a prospective study to explore the combination of tropisetron and sufentanil in the PCA may provide promising results for treating postoperative rebound pain, anxiety, nausea, and vomiting concomitantly after peripheral nerve block.

## 5. Conclusions

In conclusion, our study showed that tropisetron does not reduce postoperative rebound pain after peripheral nerve blocks. Our results demonstrated that preoperative pain, bone surgery, and incision length are risk factors for postoperative rebound pain, and PCA is protective against this pain. In addition, female sex and preoperative pain are risk factors for postoperative moderate to severe anxiety. Although several risk and protective factors have been identified, further research is warranted to explore additional interventions and mechanisms that can effectively manage postoperative rebound pain and anxiety, ultimately improving outcomes in patients who receive peripheral nerve blocks.

## Disclosures

The authors have no conflict of interest to declare.
